# MicroRNA Functions in Thymic Biology: Thymic Development and Involution

**DOI:** 10.3389/fimmu.2018.02063

**Published:** 2018-09-11

**Authors:** Minwen Xu, Tao Gan, Huiting Ning, Liefeng Wang

**Affiliations:** ^1^First Affiliated Hospital of Gannan Medical University, Ganzhou, China; ^2^Department of Biotechnology, Gannan Medical University, Ganzhou, China

**Keywords:** microRNA, thymic epithelial cells, thymic development, thymic involution, thymic microenvironment, thymus aging, regulatory network

## Abstract

During the entire processes of thymus organogenesis, maturation, and involution, gene regulation occurs post-transcriptionally via recently discovered microRNA (miRNA) transcripts. Numerous reports indicate that miRNAs may be involved in the construction of a normal thymic microenvironment, which constitutes a critical component to support T lymphocyte development. MiRNAs are also expressed in thymic stromal cells including thymic epithelial cells (TECs) during maturation and senescence. This review focuses on the function of miRNAs in thymic development and involution. A better understanding of these processes will provide new insights into the regulatory network of TECs and further comprehension of how genes control TECs to maintain the thymic microenvironment during thymus development and aging, thus supporting a normal cellular immune system.

## Introduction

The thymus plays a critical role in the cellular immune system by generating T lymphocytes, which are involved in anti-tumor immunity, anti-viral, and anti-intracellular infections, as well as the establishment of self-tolerance to avoid autoimmune disorders. During the entire process of thymus organogenesis, maturation, and involution, gene regulation not only occurs at the transcriptional level via transcription factors, but is also effected at the post-transcriptional level by microRNA (miRNA) transcripts. The ubiquitous and abundant existence of such small, non-protein-coding miRNAs in worms, plants, and animals plays an important role in the regulation of gene expression primarily at the post-transcriptional level by cleavage and/or translational repression of messenger RNAs. It has become evident that miRNAs control a wide range of developmental and physiological pathways including cell proliferation, differentiation, and apoptosis. Additionally, the deregulation of miRNAs can cause developmental blockage, dysregulation, or disease. Although many phenomena during thymic development and aging are unable to be simply explained by known protein-coding genes, many novel miRNAs have been identified within recent years that are expressed in the thymus. As the systemic miRNA gene profile and their functional characterization during thymic development and aging are gradually elucidated, we have adequate reason to infer that miRNAs may be involved in the construction of the normal thymic microenvironment that supports T lymphocyte development. In this review, we focus on the specific miRNAs that are involved in the thymic stroma, and how these play a role in thymic epithelial cell (TEC) development. Through understanding these roles, we can obtain new insights regarding the regulatory network in TEC maturation and senescence, and further understand how genes control TECs to maintain the thymic microenvironment during thymus development and aging. Our review aims to reveal potential genetic targets and identify possible therapeutic tools for patients with thymic developmental diseases, which may lead to novel strategies to rejuvenate the functions of an aged thymus or delay thymic aging.

## MiRNA identification and characterization

MiRNAs comprise a large group of conserved, single-stranded, non-coding, abundant, short (~21–25 nucleotide) RNAs (1, 2). They differ from small interfering RNAs (siRNAs) as they have molecular origins that derive from genomic loci whereas siRNAs are generated from exogenous RNA, such as viral infection, artificial RNA interference (RNAi), and endogenous transposon activity. A miRNA binds to a target mRNA through imperfect complementarity, generally at multiple sites, whereas a siRNA binds to a target mRNA to form an almost perfect duplex at only one site. However, the maturation of both miRNAs and siRNAs utilizes the common RNase-III processing enzyme, Dicer (3), prior to becoming single-stranded anti-sense RNA.

The first microRNA, lin-4 RNA, was identified in 1993 (4). It encodes a 22-nucleotide non-coding RNA that is imperfectly or partially complementary to seven conserved sites located in the 3′-untranslated regions of *lin-14*, a nuclear protein gene in *Caenorhabditis elegans*. Although this small RNA was overlooked for seven years as these short non-coding RNAs were considered to be non-existent beyond nematodes, this was completely changed by the discovery of the *let-7* gene in 2000. Notably,*let-7* was present not only in *C. elegans* but also in human and fly genomes. Currently, miRNAs are accepted as phylogenetically conserved genes and have been found in all metazoan genomes, with close to 1,000 miRNAs having been identified in *C. elegans, C. briggsae, Drosophila melanogaster, Arabidopsis thaliana*, rice, mouse, rat, and human to date (5–7).

MiRNAs are considered to represent novel biological regulators, as they regulate gene expression in a sequence-specific manner. Their primary role is to function as a negative genetic switch, which is involved in post-transcriptional regulation by targeting mRNAs for cleavage, translational repression, or chromatin modification (1, 8, 9). Recently, additional miRNA functions have been discovered including the control of developmental stages (10–12), hematopoietic cell lineage decisions (13–15), cellular proliferation, cell death/apoptosis (16–19), fat metabolism (20–22), neuronal patterning in nematodes (23–25), asymmetric expression in chemosensory neurons, and involvement in oncogenesis (26–29).

To effect such functions, the expression of miRNA is temporal and spatial in specific tissues. This implies the existence of different miRNAs that are expressed in the various thymus compartments, cell types, and developmental stages, and that expression patterns may differ between fetal and adult thymi.

## MiRNAs in thymocyte development

The thymus constitutes one of the most active organs in animal life. It undergoes organogenesis (cell migration, proliferation, and differentiation), development (proliferation, differentiation, and cell apoptosis), and involution (cell senescence and apoptosis). The thymus also generates T lymphocytes to support the cellular immune system. Generally, there are two main processes that interact and regulate each other during thymus development: T lymphocyte development to generate functional T cells, and stromal cell development to build up and maintain the thymic microenvironment for supporting T cell maturation, largely through TECs. Both of these processes represent stepwise or sequential pathways in development (30, 31).

Thymic involution results in marked morphological and functional changes; these mechanisms include TEC-driven programmed thymic involution and thymocyte apoptosis. Thymic involution results from multiple causes, which can easily be grouped into those arising from normal physiology including pregnancy and aging, and those from various pathophysiological mechanisms, such as infection, malnutrition, disease, and surgery. In particular, thymic aging involution comprises a physically progressive process that can be sped up by infections, autoimmune diseases, or cancer. In addition, a large category of pathophysiological changes can also lead to thymic involution, with infection representing a notable example (32–35).

Because miRNAs are involved in many important development events, it is not difficult to infer that numerous miRNAs are likely involved in regulating the many activities of TECs and thymocytes. Moreover, recent studies have shown that some miRNAs are present in the total thymus and are involved in T or B lymphocyte lineage determination (36–38), as well thymocyte survival. Moreover, deletion of the Dicer processing enzyme has an effect on thymocyte survival (39). Dicer promotes the development of regulatory CD4^+^ T (T reg) cells in the thymus and the efficient induction of *Foxp3* by TGF-β, whereas deletion of Dicer decreases T reg cell numbers and results in immune pathology (40). Natural T reg cells share partial overlap of miRNA expression with conventional CD4^+^ T cells. In turn, conventional CD4^+^ T cells can express CD25, CTLA4, and GITR, markers, which are also constitutively expressed by T reg cells during activation (41). Dicer deletion can also result in a distinct reduction of invariant natural killer T (iNKT) cells in the thymus and other organs with immune functions, which indicates that the Dicer-dependent miRNA pathway plays a critical role in iNKT cell development, function, and homeostasis (42–44).

Two prominent examples of miRNAs expressed in the thymus are miR-181 and miR-150. MiR-181 is highly expressed in double positive (DP) thymocytes and controls the development of early thymocyte cells by targeting CD69 and TCR (45, 46). MiR-181a, a member of the miR-181 family, controls the development of early thymocyte cells by regulating and controlling the negative feedback loops that establish the NOTCH1 and TCR signaling pathway thresholds (47, 48). In particular, these thresholds play important roles in thymic T-cell positive and negative selection, with deletion of miR-181a leading to a decrease of the early thymic progenitor cells, DN3, DP, and single positive (SP) (47). MiR-181a deletion also impairs the development of invariant αβ NK-T cells, which are agonist-selected at the DP stage (49, 50); however, miR-181a-1/b-1 is not critically required for the innate development of γδ NKT cells or any other γδ T cell subtypes (51, 52).

In comparison, miR-150 can target *c-Myb* and plays an important role in lymphocyte development and physiology (53). In human T lymphocytes, miR-150 is obviously up-regulated during T cell maturation after the DP stage and targets Notch3, which plays an important role in T cell development (54). Over-expression of miR-150 can reduce the number of T cell lines *in vitro* by impacting their proliferation and survival. MiR-150 is also expressed in iNKT cells and targets *c-Myb* (55). MiR-150 over-expression increases iNKT maturation whereas deletion of the miRNA results in an interruption of iNKT cell final maturation in both the thymus and the peripheral space (56).

In addition, some other miRNAs, such as miR-155 (57–59), miR-19b (60), let-7 (61), and miR-17 (62), have been reported to play important roles in lymphocyte maturation, differentiation, development, and survival. The roles of certain miRNA candidates in thymocyte biology are listed in Table 1.

**Table 1:** The role of candidate miRNAs in thymocyte biology.

**miRNAs**	**Cell type**	**Biologic role**	**Targets**	**References**
miR-150	T cell	Maturation of T cells	NOTCH3	(54)
	NKT/iNKT	Development of NKT ↑ iNKT ↓	C-Myc	(55, 56)
miR-155	iNKT	Maturation and differentiation of iNKT ↓	Ets1, ITK	(57)
	Treg	Development of Tregs ↑	Foxp3	(58, 59)
miR-181a-1/b-1	T cell Leukemia cell	Development	NOTCH1	(47)
	iNKT	Development of iNKT ↑	Ptpn22, Shp-2, Dusp6	(49)
miR-181	NKT	Maturation of NKT	PTEN	
miR-181a	T cell	T cell sensitivity and selection		(48)
miR-181d	CD4^+^CD8^+^	Immature CD4^+^CD8^+^ ↓	Foxo4, Myc	(52)
miR-19b	Th17	Development of Th17	Tslp	(60)
let-7	NKT		Zbtb16	(61)
miR-17	T cell	Survival	Jak1	(62)

## MiRNAs in TEC biology

As described above, TECs have three maturation stages, which can be segregated according to cell surface molecules (63). In wild type thymus, TECs completely differentiate into the three-dimensional cortical and medullar network TEC system. However, in the various stages of lymphocyte development identified by mutating the thymus, TECs themselves are arrested at different stages, indicating that TEC differentiation is tightly dependent on T-cell development. For example, in the thymus of mice with an Ikaros-null mutation (64) or the *RAG2*/common chain compound gene knock-out mutant thymus (64–66), which display distinct defects in the development of fetal and adult lymphocytes, the TECs are arrested during the early two-dimensional cortical TEC stage (67), whereas in the *RAG* null thymus, TECs are arrested in the middle three-dimensional stage.

As the expression of miRNAs is tightly regulated during tissue differentiation (68) and miRNAs can function to prevent cell division and drive terminal differentiation (69), miRNAs are therefore likely to be involved in thymic differentiation. Consistent with this supposition, a role of miRNAs in TEC biology has been demonstrated. In particular, miRNA microarray analysis of cortical thymic epithelial cells (cTECs) along with immature medullary thymic epithelial cell (mTEC)^low^ and mature mTEC^high^ cells indicated that miRNA expression differs among thymic cell subsets and fluctuates during TEC maturation (70). When Dicer was conditionally deleted in all TECs, thymus cellularity was decreased and the thymus failed to maintain a regular microenvironment (71). Moreover, mTEC apoptosis was enhanced in these mice, in which cTEC failed to impose efficient positive selection, T cell phenotypes were changed, and T lymphopoietic activity was decreased (71, 72). To further clarify the function of canonical miRNAs in TECs, *DGCR8*, encoding a component of the miRNA-specific microprocessor complex, was deleted (73). DGCR8 is critical for maintaining the proper expression of Aire, the gene for which is specifically expressed in the TEC compartment and affects TEC function, along with the overall architecture of the thymic medulla. Furthermore, miRNA deficiency in TECs causes a breakdown in central tolerance (73).

## MiRNAs in thymic involution

Although the mechanism of thymic involution remains unclear, certain miRNAs have been reported to be involved in thymic aging involution. Microarray data analysis shows that some microRNAs are significantly changed in aged thymuses, with quantitative polymerase chain reaction (qPCR) data confirming these changes (74). In particular, miR-181a-5p has been hypothesized to be associated with thymic aging involution as its expression is obviously decreased in TECs from aged mice. To test this hypothesis, a miR-181a-5p mimic was used in a mouse mTEC cell line (MTEC1). The miR-181a-5p mimic could induce cell proliferation of MTEC1 whereas its inhibitor reversed this effect. MiR-181a-5p was shown to target transforming growth factor beta receptor (*Tgfbr1*) gene using a luciferase reporter assay (75). Furthermore, the miR-181a-5p mimic could decrease Tgfbr1 protein expression as well as that of p-Smad3, is a key node of the TGF-β signaling pathway, *in vitro*. Tgfbr1 expression increases with age in mice, which is consistent with the decreased level of miR-181a-5p in addition to the ability of TGF-β to decrease the proliferation of mTECs.

In comparison, FoxN1 constitutes a pivotal transcription factor for TEC survival and differentiation, which decreases with age. WNT signaling in thymic epithelia is essential for normal thymus development and function (76) but is suppressed in the senescent human thymus (77). WNT4 can directly up-regulate FoxN1, indicating that miRNAs that target *FoxN1* or the WNT signaling pathway may be involved in thymic aging involution (78). Consistent with this, a study comparing the difference in miRNA expression between old and young thymi (from 70-year-old men vs. <10-month-old newborns, respectively) found that some miRNAs that act as modulators of the WNT pathway, such as miR-25, miR-7f, and miR-134, were among those altered (79).

Moreover, in a previous study from our laboratory, we compared changes in miRNA expression profiles between young and aged TECs using miRBase-V20 arrays (containing 1,892 unique probes), which clearly identified and validated that at least one miRNA, miR-125a-5p, was increased in aged thymi (80). In addition, the application of a miR-125a-5p mimic was able to inhibit FoxN1 expression (as indicated using 3′UTR luciferase activity) in a 293T cell line and suppress *FoxN1* expression in TEC Z210 cells (80).

The thymus represents an organ that is hyper-responsive to stress in the form of infections, radiation exposure, trauma, and drugs. Infection can induce a rapid yet transient atrophy, which is distinct from thymic aging involution. Such atrophy can recur after exposure to pathogen-associated molecular patterns (PAMPs) (81).

There is mounting evidence that miRNA expression is associated with stress. Some miRNAs might serve as potential biomarkers of stress specifically in the thymus: for example, the expression of miR-21 is increased during radiation-induced thymic lymphoma and its expression could be induced by the TGF-β (82) and by the TLR4 pathway (83). MiR-23a/b is also up-regulated in radiation-induced thymic lymphoma (84). MiRNAs may also play significant roles in protective mechanisms for counteracting stress. In particular, miR-34a and miR-7 may counteract radiation cytotoxicity by respectively targeting *NOTCH1, MYC, E2F3*, cyclin D1, and lymphoid-specific helicase (*LSH*) (85).

Alternatively, some miRNAs may play a reverse role. For example, miR-467a directly targets *Fas* and/or *Bax* and may have oncogenic functions in radiation-induced thymic lymphoma (86). Together, the evidence suggests that some miRNAs might serve as new biomarkers of stress-induced thymic injury or as novel therapeutic targets of stress-induced thymic injury. Moreover, some miRNAs might be suitable for use as drugs to treat stress-induced thymic injury. The potential roles of candidate miRNAs in thymic biology are listed in Table 2.

**Table 2:** The role of candidate miRNAs in thymic biology.

**miRNAs**	**Organism status/biologic role**	**Targets**	**References**
miR-181a-5p	Aging involution	smad3	(75)
miR-125a-5p	Aging involution	FoxN1	(80)
miR-25	Aging involution	WNT	(79)
miR-7f			
miR-134			
miR-29a	Infection	Ifnar1	(73)
miR-205	Inflammatory	FoxN1	
miR-182	Toxicity	AhR, CYP1A1, Fas, FasL	
miR-31			
miR-23a			
miR-18b			
mmu-let-7e			
miR-34a	IR-inducible involution	NOTCH1, MYC, E2F3, Cyclin D1	(85)
miR-7		LSH	
miR-21	IR-inducible involution	Big-h3	(82, 83)
miR-27b	IR-inducible involution	Cyclin A2	
miR-23a/b	IR-inducible thymic lymphoma	Fas	(84)
miR-23a/b	IR-inducible thymic lymphoma	Fas/Bax	(86)

## MiRNAs directly target signal pathway genes or *vice versa*

The adult thymic microenvironment consists of epithelial cells, fibroblastoid cells, dendritic cells, and macrophages. Epithelial cells represent the resident cell type of the thymic microenvironment. Their development and differentiation depend on a variety of signaling pathways, such as WNT (87), tumor necrosis factor receptor (TNFR) and the downstream NF-κB (88), BMP (89), IFNAR1 pathway (72), and TGF-beta (90) signaling. Interferon (IFN)-α, a critical molecular mediator of pathogen-induced thymic involution, mediates rapid and transient involution by binding IFNAR1, which is expressed on the thymic stroma (81). The Dicer-dependent miRNA network, and specifically miR-29a, is critical for reducing the sensitivity of the thymic epithelium to simulated infection signals, protecting the thymus against infection-associated thymic involution. Loss of Dicer or the miR-29a cluster in the thymic epithelium results in IFNAR1-dependent hypersensitivity to pathogen-related signals, thereby allowing suboptimal signals to trigger the rapid loss of thymic cellularity (91).

TGF-β signaling might also play an important role in controlling thymus development and maintenance (92), especially by increasing the size of the mTEC compartment and enhancing negative selection and functional maturation of medullary thymocytes (93). The TGF-β pathway components, such as receptors or transcription factors, might thus serve as targets of miRNAs. Consistent with this, TGF-β receptor 1 was confirmed as a direct target of miR-181a-5p by luciferase assay (75). Over expression of miR-181a-5p down-regulated the phosphorylation of Smad3 and blocked the activation of TGF-β signaling. In turn, Smad7, which functions as a regulator of the TGF-β signaling pathway by preventing the phosphorylation of Smad2/3, was confirmed as a direct target of miR-195a-5p. Notably, miR-195a-5p is up-regulated in mouse TECs and over-expression of miR-195a-5p inhibits the expression of TEC cell cycle-related genes including those encoding cyclin D1, cyclin E1, Cdk4, and C-myc by down-regulating the expression of Smad7 (94).

The TNFR and NF-κB pathway constitutes another important pathway for mTEC development, which is required to successfully establish the medullary microenvironment (88). Specifically, mice deficient for receptor activator of NF-κB (RANK) exhibit variable defects in mTECs (95). MiRNAs also participate in this signaling pathway and regulate mTEC differentiation. For example, RANK ligand and downstream canonical or non-canonical NF-κB can induce the expression of miR-449a. In turn, overexpression of miR-449a as well as miR-34a, which shares similar seed sequence with miR-449a, may induce TEC differentiation *in vitro* by targeting SATB2 (an epigenetic regulator identified as an miRN-449a target in colorectal tumor cells (96), as SATB2 was significantly decreased in a thymic epithelial progenitor cell line following miR-449a overexpression (91).

## MiRNAs directly target transcription factor genes of thymic stromal cell homeostasis

Transcription factors Foxn1 and p63 also play crucial roles in thymic biology. Foxn1 has an important function in TEC survival and differentiation by promoting thymic epithelial progenitor cells to differentiate into functional mTECs and cTECs during organogenesis (97, 98) and for postnatal TEC homeostasis (99, 100). p63 is important for the development of the thymus (101) and is essential for the proliferative potential of thymic epithelial progenitor cells (101, 102). Several reports have shown that miRNAs participate in TEC development and differentiation by directly targeting the *Foxn1* gene. For example, Kushwaha et al., screened out two miRNAs, miR-18b and miR-518b, that directly targeted Foxn1 (103). Their results demonstrate that miR-18b and miR-518b act as upstream controllers of Foxn1 in epithelial cell development. Moreover, interfering with these miRNAs individually or together can up-regulate Foxn1 gene expression whereas their individual or combined over-expression can decrease Foxn1 protein levels. In turn, miR-22 also regulates epithelial cell development via direct inhibition of Foxn1 (104).

p63 also serves as a target of numerous miRNAs. 29MiR-203 has immediate and long-term impact on epidermal cell proliferation by directly regulating p63 (105–107). The regulation of p63 by IASPP, an inhibitory member of the apoptosis stimulating protein of p53 (ASPP) family, via miR-574-3p and miR-720 is required for epithelial homeostasis (108). Notably, p63-mediated cell cycle progression in epidermal cells occurs through the direct repression of miR-34a and miR-34c (109). Furthermore, several miRNAs, such as miR-192/215, miR-107, miR-96,132, and miR-145, are known transcriptional targets of p63. In particular, the role of the p63-FoxN1 regulatory axis in the regulation of postnatal TEC homeostasis has been revealed by Burnley et al. (110), Overall, miRNA function can be defined as having a fine-tuning effect by targeting the p63-FoxN1 regulatory axis.

Aire constitutes another transcription factor that controls peripheral tissue-restricted antigens in mTECs. miR-29a deletion resulted in a progressive decrease in expression of *Aire* and Aire-dependent genes in miR-29a null mutant mice (70). In addition, miR-220b may act as a possible regulatory factor for *Aire* gene translation as it could significantly reduce the expression of Aire protein (111).

## Perspectives

MiRNAs play important roles in the processes of thymus organogenesis, maturation, and involution at a post-transcriptional level by targeting relevant mRNAs. Herein, we reviewed some of the miRNAs involved in thymocyte development, thymic architecture, thymic aging involution, and thymic involution during stress. We hope this review will help to deepen the appreciation of miRNA impact on thymic biology and facilitate the identification of potential candidates for therapeutic targeting. In addition, we checked miRNA profiles of serum-derived exosomes from young and aged mice with microarray of *Mus musculus* miRBase version-21 array chips and we found that young and old showed different miRNA expression profiles (112). These different spectrums of microRNAs in the young and old exosomes may generate a base for a potential epigenetic regulation and may play important roles in the processes of thymus organogenesis, maturation, and involution.

## Author contributions

MX and LW wrote the paper. TG and HN reviewed and edited the manuscript. All authors read and approved the manuscript.

### Conflict of interest statement

The authors declare that the research was conducted in the absence of any commercial or financial relationships that could be construed as a potential conflict of interest.

## Abbreviations

Aire: autoimmune regulator
cTEC: cortical thymic epithelial cell
DP: double positive
iNKT: invariant natural killer T cell
IR: ionizing radiation
miRNA: microRNA
mTEC: medullary thymic epithelial cell
TEC: thymic epithelial cell.

## References

[1] Bartel DP. MicroRNAs: genomics, biogenesis, mechanism, and function. Cell (2004) 116:281–97. 10.1016/S0092-8674(04)00045-5 14744438

[2] Lee Y Kim M Han J Yeom KH Lee S Baek SH. MicroRNA genes are transcribed by RNA polymerase II. EMBO J. (2004) 23:4051–60. 10.1038/sj.emboj.7600385 15372072 PMC524334

[3] Ketting RF Fischer SE Bernstein E Sijen T Hannon GJ Plasterk RH. Dicer functions in RNA interference and in synthesis of small RNA involved in developmental timing in *C. elegans*. Genes Dev. (2001) 15:2654–9. 10.1101/gad.927801 11641272 PMC312808

[4] Lee RC Feinbaum RL Ambros V. The *C. elegans* heterochronic gene lin-4 encodes small RNAs with antisense complementarity to lin-14. Cell (1993) 75:843–54. 8252621 10.1016/0092-8674(93)90529-y

[5] Griffiths-Jones S. The microRNA Registry. Nucleic Acids Res. (2004) 32:D109–11. 10.1093/nar/gkh023 14681370 PMC308757

[6] He L Hannon GJ. MicroRNAs: small RNAs with a big role in gene regulation. Nat Rev Genet. (2004) 5:522–31. 10.1038/nrg1379 15211354

[7] Bentwich I Avniel A Karov Y Aharonov R Gilad S Barad O. Identification of hundreds of conserved and nonconserved human microRNAs. Nat Genet. (2005) 37:766–70. 10.1038/nrg1590 15965474

[8] Pepin G Gantier MP. MicroRNA decay: Refining microRNA regulatory activity. Microrna (2016) 5:167–74. 10.2174/2211536605666161027165915 27804865

[9] Ambros V. The functions of animal microRNAs. Nature (2004) 431:350–5. 10.1038/nature02871 15372042

[10] Leong JW Sullivan RP Fehniger TA. MicroRNA management of NK-cell developmental and functional programs. Eur J Immunol. (2014) 44:2862–8. 10.1002/eji.201444798 25142111 PMC4228684

[11] Zhang H Artiles KL Fire AZ. Functional relevance of “seed” and “non-seed” sequences in microRNA-mediated promotion of *C. elegans* developmental progression. RNA (2015) 21:1980–92. 10.1261/rna.053793.115 26385508 PMC4604436

[12] Constantin L Constantin M Wainwright BJ. MicroRNA biogenesis and Hedgehog-Patched signaling cooperate to regulate an important developmental transition in granule cell development. Genetics (2016) 202:1105–18. 10.1534/genetics.115.184176 26773048 PMC4788112

[13] Chen S Wang Z Dai X Pan J Ge J Han X. Re-expression of microRNA-150 induces EBV-positive Burkitt lymphoma differentiation by modulating c-Myb *in vitro*. Cancer Sci. (2013) 104:826–34. 10.1111/cas.12156 23521217 PMC7657193

[14] Chen MT Lin HS Shen C Ma YN Wang F Zhao HL PU.1-regulated long noncoding RNA lnc-MC controls human monocyte/macrophage differentiation through interaction with microRNA 199a-5p. Mol Cell Biol. (2015) 35:3212–24. 10.1128/MCB.00429-15 26149389 PMC4539372

[15] Georgantas RW III Hildreth R Morisot S Alder J Liu CG Heimfeld S. CD34+ hematopoietic stem-progenitor cell microRNA expression and function: a circuit diagram of differentiation control. Proc Natl Acad Sci USA. (2007) 104:2750–5. 10.1073/pnas.0610983104 17293455 PMC1796783

[16] Mobarra N Shafiee A Rad SM Tasharrofi N Soufi-Zomorod M Hafizi M. Overexpression of microRNA-16 declines cellular growth, proliferation and induces apoptosis in human breast cancer cells. In Vitro Cell Dev Biol Anim. (2015) 51:604–11. 10.1007/s11626-015-9872-4 25672252

[17] Li Y Chen D Jin L Liu J Su Z Gui Y. MicroRNA-20b-5p functions as a tumor suppressor in renal cell carcinoma by regulating cellular proliferation, migration and apoptosis. Mol Med Rep. (2016) 13:1895–901. 10.3892/mmr.2015.4692 26708577

[18] Li Y Chen D Jin LU Liu J Su Z Qi Z. Oncogenic microRNA-142-3p is associated with cellular migration, proliferation and apoptosis in renal cell carcinoma. Oncol Lett. (2016) 11:1235–41. 10.3892/ol.2015.4021 26893725 PMC4734216

[19] Lenkala D LaCroix B Gamazon ER Geeleher P Im HK Huang RS. The impact of microRNA expression on cellular proliferation. Hum Genet. (2014) 133:931–8. 10.1007/s00439-014-1434-4 24609542 PMC4677487

[20] Xu P Vernooy SY Guo M Hay BA. The *Drosophila* microRNA Mir-14 suppresses cell death and is required for normal fat metabolism. Curr Biol. (2003) 13:790–5. 10.1016/S0960-9822(03)00250-1 12725740

[21] Meerson A Traurig M Ossowski V Fleming JM Mullins M Baier LJ. Human adipose microRNA-221 is upregulated in obesity and affects fat metabolism downstream of leptin and TNF-alpha. Diabetologia (2013) 56:1971–9. 10.1007/s00125-013-2950-9 23756832 PMC3737431

[22] Benatti RO Melo AM Borges FO Ignacio-Souza LM Simino LA Milanski M. Maternal high-fat diet consumption modulates hepatic lipid metabolism and microRNA-122 (miR-122) and microRNA-370 (miR-370) expression in offspring. Br J Nutr. (2014) 111:2112–22. 10.1017/S0007114514000579 24666709

[23] Johnston RJ Hobert O. A microRNA controlling left/right neuronal asymmetry in *Caenorhabditis elegans*. Nature (2003) 426:845–9. 10.1038/nature02255 14685240

[24] Hsieh YW Chang C Chuang CF. The microRNA mir-71 inhibits calcium signaling by targeting the TIR-1/Sarm1 adaptor protein to control stochastic L/R neuronal asymmetry in *C. elegans*. PLoS Genet. (2012) 8: e1002864. 10.1371/journal.pgen.1002864 22876200 PMC3410857

[25] Chang S Johnston RJ Jr Frøkjaer-Jensen C Lockery S Hobert O. MicroRNAs act sequentially and asymmetrically to control chemosensory laterality in the nematode. Nature (2004) 430:785–9. 10.1038/nature02752 15306811

[26] Sun L Wang Q Gao X Shi D Mi S Han Q. MicroRNA-454 functions as an oncogene by regulating PTEN in uveal melanoma. FEBS Lett. (2015) 589:2791–6. 10.1016/j.febslet.2015.08.007 26296312

[27] Krause CJ Popp O Thirunarayanan N Dittmar G Lipp M Müller G. MicroRNA-34a promotes genomic instability by a broad suppression of genome maintenance mechanisms downstream of the oncogene KSHV-vGPCR. Oncotarget (2016) 7:10414–32. 10.18632/oncotarget.7248 26871287 PMC4891129

[28] Bueno MJ Pérez de Castro I Gómez de Cedrón M Santos J Calin GA Cigudosa JC. Genetic and epigenetic silencing of microRNA-203 enhances ABL1 and BCR-ABL1 oncogene expression. Cancer Cell (2016) 29:607–8. 10.1016/ccr.2008.04.018 27070707

[29] Fukumoto I Koshizuka K Hanazawa T Kikkawa N Matsushita R Kurozumi A. The tumor-suppressive microRNA-23b/27b cluster regulates the MET oncogene in oral squamous cell carcinoma. Int J Oncol. (2016) 49:1119–29. 10.3892/ijo.2016.3602 27573718

[30] Klug DB Carter C Crouch E Roop D Conti CJ Richie ER. Interdependence of cortical thymic epithelial cell differentiation and T-lineage commitment. Proc Natl Acad Sci USA. (1998) 95:11822–7. 9751749 10.1073/pnas.95.20.11822 PMC21724

[31] van Ewijk W Holländer G Terhorst C Wang B. Stepwise development of thymic microenvironments *in vivo* is regulated by thymocyte subsets. Development (2000) 127:1583–91. 10725235 10.1242/dev.127.8.1583

[32] Szeri I Anderlik P Bános Z Wessely M. Bacterial modulation of the cellular immune response in mice. I. The course of lymphocytic choriomeningitis virus infection in *Bordetella pertussis* vaccine pretreated mice with physiological thymus involution. Acta Microbiol Hung. (1983) 30:227–32. 6675411

[33] Knobler RL Oldstone MB. Infection and involution of mouse thymus by MHV-4. Adv Exp Med Biol. (1987) 218:451–3. 2829559 10.1007/978-1-4684-1280-2_56

[34] Szeri I Anderlik P Bános Z Barna Z. [Effect of microbial immunomodulators on the course of lymphocytic choriomeningitis virus infection in old mice with physiological thymus involution]. Acta Pharm Hung. (1991) 61:246–52. 1785354

[35] Szeri I Anderlik P Bános Z Barna Z. Effect of microbial immunomodulants on the course of LCMV infection in old mice with thymus involution. Acta Microbiol Hung. (1992) 39:13–9. 1632194

[36] Bartel DP Chen CZ. Micromanagers of gene expression: the potentially widespread influence of metazoan microRNAs. Nat Rev Genet. (2004) 5:396–400. 10.1038/nrg1328 15143321

[37] Barad O Meiri E Avniel A Aharonov R Barzilai A Bentwich I. MicroRNA expression detected by oligonucleotide microarrays: system establishment and expression profiling in human tissues. Genome Res. (2004) 14:2486–94. 10.1101/gr.2845604 15574827 PMC534673

[38] Monticelli S Ansel KM Lee DU Rao A. Regulation of gene expression in mast cells: micro-rNA expression and chromatin structural analysis of cytokine genes. Novartis Found Symp. (2005) 271:179–87. 16605135

[39] Cobb BS Nesterova TB Thompson E Hertweck A O'Connor E Godwin J. T cell lineage choice and differentiation in the absence of the RNase III enzyme Dicer. J Exp Med. (2005) 201:1367–73. 10.1084/jem20050572 15867090 PMC2213187

[40] Muljo SA Ansel KM Kanellopoulou C Livingston DM Rao A Rajewsky K. Aberrant T cell differentiation in the absence of Dicer. J Exp Med. (2005) 202:261–9. 10.1084/jem20050678 16009718 PMC2212998

[41] Cobb BS Hertweck A Smith J O'Connor E Graf D Cook T. A role for Dicer in immune regulation. J Exp Med. (2006) 203:2519–27. 10.1084/jem.20061692 17060477 PMC2118134

[42] Fedeli M Napolitano A Wong MP Marcais A de Lalla C Colucci F. Dicer-dependent microRNA pathway controls invariant NKT cell development. J Immunol. (2009) 183:2506–12. 10.4049/jimmunol.0901361 19625646

[43] Zhou L Seo KH He HZ Pacholczyk R Meng DM Li CG. Tie2cre-induced inactivation of the miRNA-processing enzyme Dicer disrupts invariant NKT cell development. Proc Natl Acad Sci USA. (2009) 106:10266–71. 10.1073/pnas.0811119106 19509335 PMC2700920

[44] Seo KH Zhou L Meng D Xu J Dong Z Mi QS. Loss of microRNAs in thymus perturbs invariant NKT cell development and function. Cell Mol Immunol. (2010) 7:447–53. 10.1038/cmi.2010.49 20852654 PMC4002964

[45] Ebert PJ Jiang S Xie J Li QJ Davis MM. An endogenous positively selecting peptide enhances mature T cell responses and becomes an autoantigen in the absence of microRNA miR-181a. Nat Immunol. (2009) 10:1162–9. 10.1038/ni.1797 19801983 PMC3762483

[46] Liu J Wu CP Lu BF Jiang JT. Mechanism of T cell regulation by microRNAs. Cancer Biol Med. (2013) 10:131–7. 10.7497/j.issn.2095-3941.2013.03.002 24379987 PMC3860337

[47] Fragoso R Mao T Wang S Schaffert S Gong X Yue S. Modulating the strength and threshold of NOTCH oncogenic signals by mir-181a-1/b-1. PLoS Genet. (2012) 8:e1002855. 10.1371/journal.pgen.1002855 22916024 PMC3415433

[48] Li QJ Chau J Ebert PJ Sylvester G Min H Liu G. miR-181a is an intrinsic modulator of T cell sensitivity and selection. Cell (2007) 129:147–61. 10.1016/j.cell.2007.03.008 17382377

[49] Henao-Mejia J Williams A Goff LA Staron M Licona-Limón P Kaech SM. The microRNA miR-181 is a critical cellular metabolic rheostat essential for NKT cell ontogenesis and lymphocyte development and homeostasis. Immunity (2013) 38:984–97. 10.1016/j.immuni.2013.02.021 23623381 PMC3738211

[50] Zietara N Lyszkiewicz M Witzlau K Naumann R Hurwitz R Langemeier J. Critical role for miR-181a/b-1 in agonist selection of invariant natural killer T cells. Proc Natl Acad Sci USA. (2013) 110:7407–12. 10.1073/pnas.1221984110 23589855 PMC3645533

[51] Sandrock I Zietara N Łyszkiewicz M Oberdörfer L Witzlau K Krueger A MicroRNA-181a/b-1 is not required for innate γδ NKT effector cell development. PLoS ONE (2015) 10:e0145010 10.1371/journal.pone.0145010 26673421 PMC4682956

[52] Belkaya S van Oers NS. Transgenic expression of microRNA-181d augments the stress-sensitivity of CD4(+)CD8(+) thymocytes. PLoS ONE (2014) 9:e85274. 10.1371/journal.pone.0085274 24416377 PMC3887031

[53] Zhou B Wang S Mayr C Bartel DP Lodish HF. miR-150, a microRNA expressed in mature B and T cells, blocks early B cell development when expressed prematurely. Proc Natl Acad Sci USA. (2007) 104:7080–5. 10.1073/pnas.0702409104 17438277 PMC1855395

[54] Ghisi M Corradin A Basso K Frasson C Serafin V Mukherjee S. Modulation of microRNA expression in human T-cell development: targeting of NOTCH3 by miR-150. Blood (2011) 117:7053–62. 10.1182/blood-2010-12-326629 21551231

[55] Bezman NA Chakraborty T Bender T Lanier LL. miR-150 regulates the development of NK and iNKT cells. J Exp Med. (2011) 208:2717–31. 10.1084/jem.20111386 22124110 PMC3244033

[56] Zheng Q Zhou L Mi QS. MicroRNA miR-150 is involved in Valpha14 invariant NKT cell development and function. J Immunol. (2012) 188:2118–26. 10.4049/jimmunol.1103342 22287707 PMC7375412

[57] Burocchi A Pittoni P Tili E Rigoni A Costinean S Croce CM. Regulated expression of miR-155 is required for iNKT cell development. Front Immunol. (2015) 6:140. 10.3389/jimmu.2015.00140 25870598 PMC4378312

[58] Kohlhaas S Garden OA Scudamore C Turner M Okkenhaug K Vigorito E. Cutting edge: the Foxp3 target miR-155 contributes to the development of regulatory T cells. J Immunol. (2009) 182:2578–82. 10.4049/jimmunol.0803162 19234151

[59] Stahl HF Fauti T Ullrich N Bopp T Kubach J Rust W. miR-155 inhibition sensitizes CD4+ Th cells for TREG mediated suppression. PLoS ONE (2009) 4:e7158. 10.1371/journal.pone.0007158 19777054 PMC2743997

[60] Wang Z Chen Y Xu S Yang Y Wei D Wang W. Aberrant decrease of microRNA19b regulates TSLP expression and contributes to Th17 cells development in myasthenia gravis related thymomas. J Neuroimmunol. (2015) 288:34–9. 10.1016/j.jneuroim.2015.08.013 26531692

[61] Pobezinsky LA Etzensperger R Jeurling S Alag A Kadakia T McCaughtry TM. Let-7 microRNAs target the lineage-specific transcription factor PLZF to regulate terminal NKT cell differentiation and effector function. Nat Immunol. (2015) 16:517–24. 10.1038/ni.3146 25848867 PMC4406853

[62] Katz G Pobezinsky LA Jeurling S Shinzawa M Van Laethem F Singer A. T cell receptor stimulation impairs IL-7 receptor signaling by inducing expression of the microRNA miR-17 to target Janus kinase 1. Sci Signal. (2014) 7:ra83. 10.1126/scisignal.2005221 25161318 PMC6679598

[63] Takahama Y Ohigashi I Baik S Anderson G. Generation of diversity in thymic epithelial cells. Nat Rev Immunol. (2017) 17:295–305. 10.1038/nri.2017.12 28317923

[64] Wang JH Nichogiannopoulou A Wu L Sun L Sharpe AH Bigby M. Selective defects in the development of the fetal and adult lymphoid system in mice with an Ikaros null mutation. Immunity (1996) 5:537–49. 8986714 10.1016/s1074-7613(00)80269-1

[65] Leonard WJ Shores EW Love PE. Role of the common cytokine receptor gamma chain in cytokine signaling and lymphoid development. Immunol Rev. (1995) 148:97–114. 8825284 10.1111/j.1600-065x.1995.tb00095.x

[66] Colucci F Soudais C Rosmaraki E Vanes L Tybulewicz VL Di Santo JP. Dissecting NK cell development using a novel alymphoid mouse model: investigating the role of the c-abl proto-oncogene in murine NK cell differentiation. J Immunol. (1999) 162:2761–5. 10072522

[67] Klug DB Carter C Gimenez-Conti IB Richie ER. Cutting edge: thymocyte-independent and thymocyte-dependent phases of epithelial patterning in the fetal thymus. J Immunol. (2002) 169:2842–5. 10.4049/jimmunol.169.6.2842 12218095

[68] Lu J Getz G Miska EA Alvarez-Saavedra E Lamb J Peck D. MicroRNA expression profiles classify human cancers. Nature (2005) 435:834–8. 10.1038/nature03702 15944708

[69] Reinhart BJ Slack FJ Basson M Pasquinelli AE Bettinger JC Rougvie AE. The 21-nucleotide let-7 RNA regulates developmental timing in *Caenorhabditis elegans*. Nature (2000) 403:901–6. 10.1038/35002607 10706289

[70] Ucar O Tykocinski LO Dooley J Liston A Kyewski B. An evolutionarily conserved mutual interdependence between Aire and microRNAs in promiscuous gene expression. Eur J Immunol. (2013) 43:1769–78. 10.1002/eji.201343343 23589212 PMC3816332

[71] Zuklys S Mayer CE Zhanybekova S Stefanski HE Nusspaumer G Gill J. MicroRNAs control the maintenance of thymic epithelia and their competence for T lineage commitment and thymocyte selection. J Immunol. (2012) 189:3894–904. 10.4049/jimmunol.1200783 22972926 PMC3675876

[72] Papadopoulou AS Dooley J Linterman MA Pierson W Ucar O Kyewski B. The thymic epithelial microRNA network elevates the threshold for infection-associated thymic involution via miR-29a mediated suppression of the IFN-alpha receptor. Nat Immunol. (2011) 13:181–7. 10.1038/ni.2193 22179202 PMC3647613

[73] Khan IS Taniguchi RT Fasano KJ Anderson MS Jeker LT. Canonical microRNAs in thymic epithelial cells promote central tolerance. Eur J Immunol. (2014) 44:1313–9. 10.1002/eji.201344079 24515814 PMC4141217

[74] Guo Z Chi F Song Y Wang C Yu R Wei T. Transcriptome analysis of murine thymic epithelial cells reveals ageassociated changes in microRNA expression. Int J Mol Med. (2013) 32:835–42. 10.3892/ijmm.2013.1471 23969555

[75] Guo D Ye Y Qi J Zhang L Xu L Tan X. MicroRNA-181a-5p enhances cell proliferation in medullary thymic epithelial cells via regulating TGF-beta signaling. Acta Biochim Biophys Sin. (2016) 48:840–9. 10.1093/abbs/gmw068 27411504

[76] Zuklys S Gill J Keller MP Hauri-Hohl M Zhanybekova S Balciunaite G. Stabilized beta-catenin in thymic epithelial cells blocks thymus development and function. J Immunol. (2009) 182:2997–3007. 10.4049/jimmunol.0713723 19234195

[77] Wang B Hollander GA Nichogiannopoulou A Simpson SJ Orange JS Gutierrez-Ramos JC. Natural killer cell development is blocked in the context of aberrant T lymphocyte ontogeny. Int Immunol. (1996) 8:939–49. 8671683 10.1093/intimm/8.6.939

[78] Talaber G Kvell K Varecza Z Boldizsar F Parnell SM Jenkinson EJ. Wnt-4 protects thymic epithelial cells against dexamethasone-induced senescence. Rejuvenation Res. (2011) 14:241–8. 10.1089/rej.2010.1110 21453014 PMC3136744

[79] Ferrando-Martinez S Ruiz-Mateos E Dudakov JA Velardi E Grillari J Kreil DP. WNT signaling suppression in the senescent human thymus. J Gerontol A Biol Sci Med Sci. (2015) 70:273–81. 10.1093/gerona/glu030 24657825 PMC4351388

[80] Xu M Sizova O Wang L Su DM. A fine-tune role of mir-125a-5p on Foxn1 during age-associated changes in the thymus. Aging Dis. (2017) 8:277–86. 10.14336/AD.2016.1109 28580184 PMC5440108

[81] Anz D Thaler R Stephan N Waibler Z Trauscheid MJ Scholz C. Activation of melanoma differentiation-associated gene 5 causes rapid involution of the thymus. J Immunol. (2009) 182:6044–50. 10.4049/jimmunol.0803809 19414755

[82] Liu C Li B Cheng Y Lin J Hao J Zhang S. MiR-21 plays an important role in radiation induced carcinogenesis in BALB/c mice by directly targeting the tumor suppressor gene Big-h3. Int J Biol Sci. (2011) 7:347–63. 10.7150/ijbs.7.347 21494432 PMC3076505

[83] Liu C Gao F Li B Mitchel RE Liu X Lin J. TLR4 knockout protects mice from radiation-induced thymic lymphoma by downregulation of IL6 and miR-21. Leukemia (2011) 25:1516–9. 10.1038/leu.2011.113 21617699

[84] Li B Sun M Gao F Liu W Yang Y Liu H. Up-regulated expression of miR-23a/b targeted the pro-apoptotic Fas in radiation-induced thymic lymphoma. Cell Physiol Biochem. (2013) 32:1729–40. 10.1159/000356607 24356489

[85] Ilnytskyy Y Zemp FJ Koturbash I Kovalchuk O. Altered microRNA expression patterns in irradiated hematopoietic tissues suggest a sex-specific protective mechanism. Biochem Biophys Res Commun. (2008) 377:41–5. 10.1016/j.bbrc.2008.09.080 18823940

[86] Gao F Chen S Sun M Mitchel RE Li B Chu Z. MiR-467a is upregulated in radiation-induced mouse thymic lymphomas and regulates apoptosis by targeting Fas and Bax. Int J Biol Sci. (2015) 11:109–21. 10.7150/jibs.10276 25552935 PMC4278260

[87] Huang A Zhang H Chen S Xia F Yang Y Dong F. miR-34a expands myeloid-derived suppressor cells via apoptosis inhibition. Exp Cell Res. (2014) 326:259–66. 10.1016/j.yexcr.2014.04.010 24780820

[88] Osada M Jardine L Misir R Andl T Millar SE Pezzano M. DKK1 mediated inhibition of Wnt signaling in postnatal mice leads to loss of TEC progenitors and thymic degeneration. PLoS ONE (2010) 5:e9062. 10.1371/journal.pone.0009062 20161711 PMC2817005

[89] Akiyama T Shimo Y Yanai H Qin J Ohshima D Maruyama Y. The tumor necrosis factor family receptors RANK and CD40 cooperatively establish the thymic medullary microenvironment and self-tolerance. Immunity (2008) 29:423–37. 10.1016/j.immuni.2008.06.015 18799149

[90] Bleul CC Boehm T. BMP signaling is required for normal thymus development. J Immunol. (2005) 175:5213–21. 10.4049/jimmunol.175.8.5213 16210626

[91] Hauri-Hohl MM Zuklys S Keller MP Jeker LT Barthlott T Moon AM. TGF-beta signaling in thymic epithelial cells regulates thymic involution and postirradiation reconstitution. Blood (2008) 112:626–34. 10.1182/blood-2007-10-115618 18474727 PMC2481556

[92] Chen P Zhang H Sun X Hu Y Jiang W Liu Z. microRNA-449a modulates medullary thymic epithelial cell differentiation. Sci Rep. (2017) 7:15915. 10.1038/s41598-017-16162-2 29162901 PMC5698406

[93] Bravo-Nuevo A O'Donnell R Rosendahl A Chung JH Benjamin LE Odaka C. RhoB deficiency in thymic medullary epithelium leads to early thymic atrophy. Int Immunol. (2011) 23:593–600. 10.1093/intimm/dxr064 21865151 PMC3182298

[94] Hauri-Hohl M Zuklys S Holländer GA Ziegler SF. A regulatory role for TGF-beta signaling in the establishment and function of the thymic medulla. Nat Immunol. (2014) 15:554–61. 10.1038/ni.2869 24728352

[95] Rossi SW Kim MY Leibbrandt A Parnell SM Jenkinson WE Glanville SH. RANK signals from CD4(+)3(-) inducer cells regulate development of Aire-expressing epithelial cells in the thymic medulla. J Exp Med. (2007) 204:1267–72. 10.1084/jem20062497 17502664 PMC2118623

[96] Sun X Liu S Chen P Fu D Hou Y Hu J. miR-449a inhibits colorectal cancer progression by targeting SATB2. Oncotarget (2017) 8:100975–88. 10.18632/oncotarget.10900 29254139 PMC5731849

[97] Nehls M Pfeifer D Schorpp M Hedrich H Boehm T. New member of the winged-helix protein family disrupted in mouse and rat nude mutations. Nature (1994) 372:103–7. 7969402 10.1038/372103a0

[98] Nehls M Kyewski B Messerle M Waldschutz R Schüddekopf K Smith AJ. Two genetically separable steps in the differentiation of thymic epithelium. Science (1996) 272:886–9. 8629026 10.1126/science.272.5263.886

[99] Cheng L Guo J Sun L Fu J Barnes PF Metzger D. Postnatal tissue-specific disruption of transcription factor FoxN1 triggers acute thymic atrophy. J Biol Chem. (2010) 285:5836–47. 10.1074/jbc.M109.072124 19955175 PMC2820809

[100] Chen L Xiao S Manley NR. Foxn1 is required to maintain the postnatal thymic microenvironment in a dosage-sensitive manner. Blood (2009) 113:567–74. 10.1182/blood-2008-05-156265 18978204 PMC2628364

[101] Candi E Rufini A Terrinoni A Giamboi-Miraglia A Lena AM Mantovani R. DeltaNp63 regulates thymic development through enhanced expression of FgfR2 and Jag2. Proc Natl Acad Sci USA. (2007) 104:11999–2004. 10.1073/pnas1713458104 17626181 PMC1924561

[102] Senoo M Pinto F Crum CP McKeon F. p63 Is essential for the proliferative potential of stem cells in stratified epithelia. Cell (2007) 129:523–36. 10.1016/j.cell.2007.02.045 17482546

[103] Kushwaha R Thodima V Tomishima MJ Bosl GJ Chaganti RS. miR-18b and miR-518b Target FOXN1 during epithelial lineage differentiation in pluripotent cells. Stem Cell Dev. (2014) 23:1149–56. 10.1089/scd.2013.0262 24383669

[104] Yuan S Li F Meng Q Zhao Y Chen L Zhang H. Post-transcriptional regulation of keratinocyte progenitor cell expansion, differentiation and hair follicle regression by miR-22. PLoS Genet. (2015) 11:e1005253. 10.1371/journal.pgen.1005253 26020521 PMC4447420

[105] Jackson SJ Zhang Z Feng D Flagg M O'Loughlin E Wang D. Rapid and widespread suppression of self-renewal by microRNA-203 during epidermal differentiation. Development (2013) 140:1882–91. 10.1242/dev.089649 23571213 PMC3631964

[106] Lena AM Shalom-Feuerstein R Rivetti di Val Cervo P Aberdam D Knight RA Melino G. miR-203 represses 'stemness' by repressing DeltaNp63. Cell Death Differ. (2008) 15:1187–95. 10.1038/cdd.2008.69 18483491

[107] Ørom UA Lim MK Savage JE Jin L Saleh AD Lisanti MP. MicroRNA-203 regulates caveolin-1 in breast tissue during caloric restriction. Cell Cycle (2012) 11:1291–5. 10.4161/cc.19704 22421148 PMC3350875

[108] Chikh A Matin RN Senatore V Hufbauer M Lavery D Raimondi C. iASPP/p63 autoregulatory feedback loop is required for the homeostasis of stratified epithelia. EMBO J. (2011) 30:4261–73. 10.1038/emboj.2011.302 21897369 PMC3199390

[109] Antonini D Russo MT De Rosa L Gorrese M Del Vecchio L Missero C. Transcriptional repression of miR-34 family contributes to p63-mediated cell cycle progression in epidermal cells. J Invest Dermatol. (2010) 130:1249–57. 10.1038/jid.2009.438 20090763

[110] Burnley P Rahman M Wang H Zhang Z Sun X Zhuge Q. Role of the p63-FoxN1 regulatory axis in thymic epithelial cell homeostasis during aging. Cell Death Dis. (2013) 4:e932. 10.1038/cddis.2013.460 24263106 PMC3847336

[111] Matsuo T Noguchi Y Shindo M Morita Y Oda Y Yoshida E. Regulation of human autoimmune regulator (AIRE) gene translation by miR-220b. Gene (2013) 530:19–25. 10.1016/j.gene.2013.08.105 23954874

[112] Wang W Wang L Ruan L Oh J Dong X Zhuge Q Extracellular vesicles extracted from young donor serum attenuate inflammaging via partially rejuvenating aged T-cell immunotolerance. FASEB J (2018). 32, 1–14. 10.1096/fj.201800059R 29782203 PMC6181631

